# High-Amylose Starch and Human Health: Microbiota Modulation, Metabolic Reprogramming, and Disease Prevention

**DOI:** 10.3390/nu18162682

**Published:** 2026-08-17

**Authors:** Md Suzauddula, Weiqun Wang, Yong-Cheng Shi

**Affiliations:** 1 School of Health Science, Kansas State University, Manhattan, KS 66506, USA; suza@ksu.edu; 2 Department of Grain and Food Science, Kansas State University, Manhattan, KS 66506, USA

**Keywords:** high-amylose starch, gut microbiota modulation, chronic disease, gut health, SCFAs, gut–brain axis

## Abstract

High-amylose starch (HAS) is composed predominantly of linear α-1,4-linked glucose units and is high in resistant starch content. Its unique physicochemical properties and gut fermentation dynamics confer multiple health benefits. This review summarizes HAS digestion, its interactions with gut microbes, and its systemic effects. HAS intake can reshape the gut microbiota by enriching beneficial taxa such as *Bifidobacterium*, *Faecalibacterium*, and *Akkermansia*, while suppressing harmful bacteria like *Escherichia coli* and *Clostridium difficile*. These microbial shifts enhance short-chain fatty acid production, improve gut barrier integrity, and reduce inflammation. HAS also demonstrates therapeutic potential in obesity, type 2 diabetes, chronic kidney disease, and colorectal cancer by improving insulin sensitivity, reducing glycemic variability, and modulating oncogenic pathways. Collectively, dietary incorporation of HAS represents a promising functional strategy to support gut and systemic health across diverse physiological contexts.

## 1. Introduction

Resistant starch (RS) is a type of starch that resists digestion in the small intestine and reaches the colon. In the colon, RS acts as a fermentable fiber and contributes to various gut health benefits. There are five types of RS: physically inaccessible starch (RS1), native granular starch (RS2), retrograded starch formed after cooking and cooling (RS3), chemically modified starch (RS4), and amylose–lipid complexes (RS5) [1]. RS2 and RS3 are often rich in amylose [2,3]. Amylose is an essentially linear polymer of glucose units linked predominately by α-1,4-glycosidic bonds, constituting 5–35% of most natural starches [4]. It is synthesized naturally in plants through the action of enzymes such as granule-bound starch synthase (GBSS), and proteins like Protein Targeting to Starch 1 (PTST1) facilitate this process by assisting with localization and GBSS function [5]. Amylose plays an important structural role in starch functionality because its relatively linear chains promote reassociation, retrogradation, and gel network formation after gelatinization. These structural characteristics also have nutritional relevance, as they can limit starch digestion in the small intestine, increase resistant starch availability, and provide a fermentable substrate for the gut microbiota in the colon [6,7,8]. Furthermore, the structure of amylose, including its molecular size and degree of branching, strongly impacts the properties and functionalities of starch in various applications [9].

Major plant sources of amylose are maize, wheat, rice, barley, potato, and various types of peas (Table 1). The amylose content of cereals, tubers, and pulses can vary significantly across varieties and genetic lines. These plant sources are extensively cultivated for their starch, which is used in both food and industrial applications.

High-amylose starch (HAS) typically contains more than 35% amylose and has attracted growing interest in both the food industry and the nutrition sector. HAS offers multifunctional health benefits and a protective effect against disease [8,43]. Evidence has emerged that HAS can alter the gut microbial profile and composition by enhancing the proliferation of beneficial bacteria. Such alteration of beneficial microorganisms is already known to be positively associated with gut health and reduced inflammation [44,45]. Among high-amylose starches, high-amylose maize starch has long been commercialized since the 1950s, and commercial RS products based on high-amylose maize starch were developed by National Starch and Chemical Corp. (later to become National Starch Food Innovation, which is now part of Ingredion Inc.). Most nutritional and health studies, including human clinical trials, used high-amylose maize starch or RS products produced by high-amylose maize starch. However, other plant sources of HAS have been developed (Table 1) largely due to their potential health benefits. Most notably, high-amylose wheat and its flour are now commercially available. High-amylose wheat flour can be directly formulated to make wheat-based foods and has great potential to offer significant health benefits due to its high RS and dietary fiber content [46]. In this review, HAS is used as a functional umbrella term for starches with higher amylose content than conventional starches from the same botanical source, but the health effects are interpreted according to the dominant mechanism of resistance rather than amylose content alone. HAS materials differ in amylose content, chain-length distribution, amylopectin branching, granule morphology, crystallinity, lipid interactions, and processing history, which can alter enzymatic resistance, colonic fermentation, and physiological outcomes (Table 2).

Because of the great interest in HAS and continued research in developing high-amylose crops, the objective of this review is to explore the role of HAS in gut health, with particular focus on its contributions to digestion, interactions with the gut microbiota, and effects on metabolic byproducts. This review examines how HAS consumption affects gut microbiota composition, promotes beneficial bacterial populations, and modulates microbial diversity. The production of secondary metabolites, such as short-chain fatty acids (SCFAs) and choline derivatives, and their implications for metabolic health are discussed. Furthermore, this review explores how modifications in the gut microbial profile contribute to gut integrity and may help prevent chronic diseases such as obesity, T2D, and colon diseases. Overall, the present review is distinct from previous publications by focusing on HAS as a microbiome-active dietary component and by integrating evidence across gut microbial remodeling, microbial metabolite production, gut barrier function, inflammation, and disease-relevant outcomes. In addition to discussing digestion and fermentation, this review summarizes how HAS may enrich beneficial bacterial taxa, suppress potentially harmful microbial groups, and influence metabolic pathways related to obesity, T2D, chronic kidney disease, colorectal cancer, and the gut–brain axis. In addition to high-amylose maize starch, the effects of HAS from other higher plants on human health are reviewed. Therefore, this review provides a broader health-oriented synthesis of HAS–microbiota–metabolite interactions rather than a review limited to starch structure, food functionality, or fermentation properties.

## 2. Digestion Process of High-Amylose Starch from the Mouth to the Colon

The digestion of starch is a complex process that consists of multiple chemical/enzymatic steps and physiological changes from the mouth to the colon (Table 3). The process is crucial for converting dietary starch into glucose, which is then absorbed and used by the body for energy. Starch digestion begins in the mouth, where salivary amylase (ptyalin) initiates starch breakdown [54]. Salivary amylase is an endo-amylase that hydrolyzes the α-1,4 glycosidic bonds in the interior of starch molecules, producing dextrins and shorter oligosaccharides such as maltose and maltotriose [55]. Differences in genetics, diet type, timing of consumption, oral cavity temperature, pH, and salivary amylase activity can vary significantly among individuals [56]. The partially hydrolyzed starch, now in the form of oligosaccharides and dextrins, is swallowed and moves to the stomach for further processing. The stomach plays a limited role in digesting starch due to its acidic environment and the absence of specific enzymes for starch breakdown. The low pH in the stomach inhibits salivary amylase activity, effectively halting its action [57]. But the stomach churns food into a semi-liquid mixture called chyme, which helps mechanically break down starch granules [58]. However, this process does not involve the enzymatic hydrolysis of starch. The primary digestion of starch happens in the small intestine, where pancreatic amylase and brush border enzymes turn starch into absorbable glucose. Any portion of starch that resists digestion in the small intestine enters the colon and is fermented by the colonic microbiota, such as Bifidobacterium, Bacteroides, and Clostridium. During bacterial fermentation, SCFAs and other useful chemicals are produced. Such byproducts have been shown to play a key role in neuro-immunoendocrine regulation [59].

Compared with general starch digestion, HAS is less completely hydrolyzed during small intestinal digestion because its structural features limit enzyme accessibility. HAS generally contains a higher resistant starch fraction and shows greater resistance to enzymatic hydrolysis than regular starch [8]. This resistance is associated with the surface features of starch granules, reduced granule swelling, crystallinity, amylose double-helical structures, amylose–lipid complexes, surface-bound proteins, reduced internal pores, and dense starch-protein or food matrix structures that restrict enzyme binding and catalytic hydrolysis [8,60]. Consequently, a larger portion of HAS escapes digestion in the upper gastrointestinal tract and reaches the colon, where it becomes available for microbial fermentation and SCFA production [8].

## 3. Impact of High-Amylose Starch on Gut Microbiota

Dietary components such as amylose alter the composition and functionality of the gut microbiota, and HAS functions as a prebiotic. Understanding how HAS affects specific bacterial populations can offer valuable insights into gut-mediated immune and metabolic regulation. The effects of different types of RS on the human gut microbiome have been studied, revealing that they alter microbiota composition in distinct ways [61]. In a short-term human dietary intervention study involving 174 healthy young adults, Baxter et al. examined gut microbiota and SCFA responses to three fermentable fibers. Hi-Maize 260 resistant starch, which is made from high-amylose maize starch, increased the relative abundance of sequences classified as *Ruminococcus bromii* 2.5-fold (*p* < 0.001), but butyrate responses varied among individuals and were influenced by baseline microbiota composition [62]. In a small double-blind crossover human trial involving 10 participants, Martínez et al. compared fecal microbiota responses to crackers containing RS2, RS4, or native wheat starch as a control. RS2 was provided as Hi-Maize 260 resistant starch, and consumption of RS2-fortified crackers significantly increased the relative abundance of *Ruminococcus bromii* and *Eubacterium rectale* compared with RS4. However, because this was a small short-term human intervention, the findings should be interpreted as evidence that RS type and chemical structure can differentially shape fecal microbiota composition rather than as definitive proof of broad clinical benefit [63]. *Bifidobacterium* is a key player in modulating host immunity by expanding populations of memory CD8+ T cells, plasmablasts, basophils, and neutrophils [64]. In an animal feeding study, Regmi et al. used eight ileal-cannulated pigs in a replicated 4 × 4 Latin-square design to compare purified starch diets containing <5%, 20%, 28%, or 63% amylose. The 63% amylose diet, which had the lowest in vitro digestibility, increased intestinal nutrient flow, microbial fermentation, and fecal *Bifidobacterium* spp. compared with lower-amylose diets, indicating a bifidogenic effect of HAS in pigs [39]. Similarly, *Lactobacillus* species contribute to gut health by reinforcing the intestinal immunological, epithelial, and mucus barriers and alleviating intestinal damage [65]. In the digesta, consumption of diets containing 50% high-amylose cornstarch increased Lactobacillus counts compared to diets without amylose [66]. Likewise, *Faecalibacterium* is an important microbe that can contribute to immune regulation. It maintains the stability of T-helper 17 (Th17) and regulatory T cells (Treg) and reduces inflammatory cytokine production by enhancing gut barrier integrity [53]. Its abundance increased 5.1-fold in response to high-amylose maize starch compared with low-amylose variants [67]. *Ruminococcus* species specialize in degrading complex carbohydrates such as cellulose, releasing nutrients that support other gut microbiota and the host; their numbers also increase with a high-amylose wheat diet [68,69,70]. Another important microbe, *Akkermansia*, helps gut immunity and barrier maintenance. Its population was markedly enriched in individuals consuming 35% high-amylose maize starch, indicating amylose’s potent prebiotic effect on beneficial gut microbiota [12,71]. These findings collectively highlight the prebiotic effect of HAS in selectively enriching health-promoting gut bacteria, suggesting that incorporating foods rich in HAS into the diet may be a strategic approach to enhance gut microbiota composition and support systemic immunity (Table 4).

Although many studies report associations between HAS intake, enrichment of taxa such as *Bifidobacterium*, *Faecalibacterium*, *Ruminococcus*, and *Akkermansia*, increased SCFA production, and improved host metabolic or inflammatory markers, these associations do not by themselves establish causality. Direct functional validation requires experimental approaches showing that HAS-induced microbial changes are necessary and/or sufficient for the observed host benefits. Evidence of this type remains limited in the HAS literature. A recent randomized, placebo-controlled, double-blind crossover human study using high-amylose maize-RS2 (HAM-RS2) in adults with overweight or obesity provided one of the strongest examples by combining human intervention data with fecal microbiota transplantation, germ-free mouse experiments, and *Bifidobacterium adolescentis* supplementation. In that study, RS-induced microbiota transfer improved obesity and glucose-related phenotypes in mice, while RS showed limited effects in germ-free mice unless *B. adolescentis* was introduced, suggesting that gut microbiota, and particularly *B. adolescentis*, partly mediated the anti-obesity effects of HAM-RS2 [73]. However, this evidence is outcome-specific and should not be generalized to all HAS sources, all microbial taxa, or all health outcomes. Therefore, in this review, HAS-induced microbiota changes are interpreted as plausible and partly supported mediators of health effects, but most taxa–health relationships remain associative unless validated by FMT, germ-free, antibiotic depletion, or microbial add-back experiments. Nevertheless, enrichment or depletion of specific taxa should be interpreted as associative evidence unless functional studies demonstrate that these microbial changes are required for, or sufficient to reproduce, the corresponding host phenotype.

## 4. Dietary HAS and Its Suppressive Effects on Harmful Gut Microbiota

HAS not only increases good gut microbiota but also selectively suppresses harmful bacterial taxa associated with gastrointestinal and systemic diseases (Table 5). For instance, pathogenic strains of *Escherichia coli* are responsible for urinary tract infections, diarrheal illness, and neonatal sepsis/meningitis. A study revealed that *Escherichia coli* was significantly reduced under HAS diets, particularly when high-amylose potato starch was used [74,75]. Similarly, *Clostridium difficile*, a major cause of nosocomial diarrhea and pseudomembranous colitis, showed a decreased representation of its clusters IV and XIVa in pigs fed diets containing 63% amylose derived from cornstarch, indicating a suppressive microbial shift [72,76]. *Salmonella* spp. is a zoonotic pathogen linked to foodborne diarrheal diseases and carcinogenesis in the colon and gallbladder. HAS has also reduced fecal abundance of *Salmonella* spp. and diminished multidrug-resistant strains in both porcine and human models [77,78,79]. Furthermore, the relative abundance of *Streptococcus* spp., which correlates with systemic inflammation and oral pathologies, was diminished in response to high-amylose cornstarch consumption [80,81]. In an in vitro anaerobic culture study, 38 human colonic bacterial strains were screened for their ability to utilize soluble starch, amylopectin maize starch, and high-amylose maize starch granules. In selected strain-level fermentation assays, *Bacteroides fragilis*, *B. vulgatus*, and *Eubacterium limosum* showed lower growth rates and poorer degradation of granular amylomaize than *Bifidobacterium* spp. and *Clostridium butyricum*. Therefore, these findings indicate substrate- and strain-specific utilization of high-amylose maize starch in vitro, but they should not be interpreted as direct evidence that HAS suppresses these taxa in vivo [82,83]. Lastly, high-amylose maize starch lowered the abundance of *Erysipelotrichaceae*, a bacterial family associated with inflammatory bowel disease and Crohn’s-like transmural inflammation [84,85]. These findings collectively support HAS as a dietary strategy to reduce pathogenic microbiota and mitigate inflammation-related gut disorders.

## 5. Health-Promoting Effects of HAS: Mechanistic Insights and Disease Prevention

### 5.1. Anti-Inflammatory Properties

HAS may be considered a prebiotic because it can promote the growth of beneficial gut microbiota. Increasing specific gut microbiota can increase the level of SCFAs, such as butyrate. For example, the resistant starch fraction of high-amylose wheat flour increased SCFAs during fermentation by the gut microbiota [86]. SCFAs are recognized for their anti-inflammatory properties [87]. In a 6-week randomized in vivo mouse feeding study, 48 six-week-old male Swiss-Webster mice were assigned to three diet groups (*n* = 16/group): normal cornstarch control, high-amylose cornstarch (HA7), or octenyl-succinate-modified high-amylose cornstarch (OS-HA7). Fecal 16S rRNA profiling showed diet-specific restructuring of the gut microbiota, and the HA7 diet enriched Actinobacteria, a bacterial class that includes *Bifidobacterium*, compared with the control diet. These findings suggest that high-amylose cornstarch can alter gut microbial composition in mice, although the study was an animal microbiota–gut–brain axis study and does not directly establish anti-inflammatory effects in humans. The anti-inflammatory relevance of *Bifidobacterium* is supported separately by in vitro macrophage screening and a DSS-induced colitis mouse model, in which selected *Bifidobacterium* strains reduced inflammatory markers and improved colitis-associated dysbiosis [88,89]. In terms of inflammatory cytokines, propionylated and non-propionylated high-amylose maize starch have been shown to decrease pro-inflammatory cytokines, namely TNF-α and IL-6, in both animal models [85]. Having selective permeability, the gut barrier prevents the translocation of unwanted and inflammatory substances into the circulation. Genetically modified high-amylose maize starch has been shown to improve gut barrier function by increasing *Akkermansia* in the gastrointestinal tract and reducing inflammatory response in vivo [12].

### 5.2. Chronic Kidney Disease (CKD)

**Figure 1:**
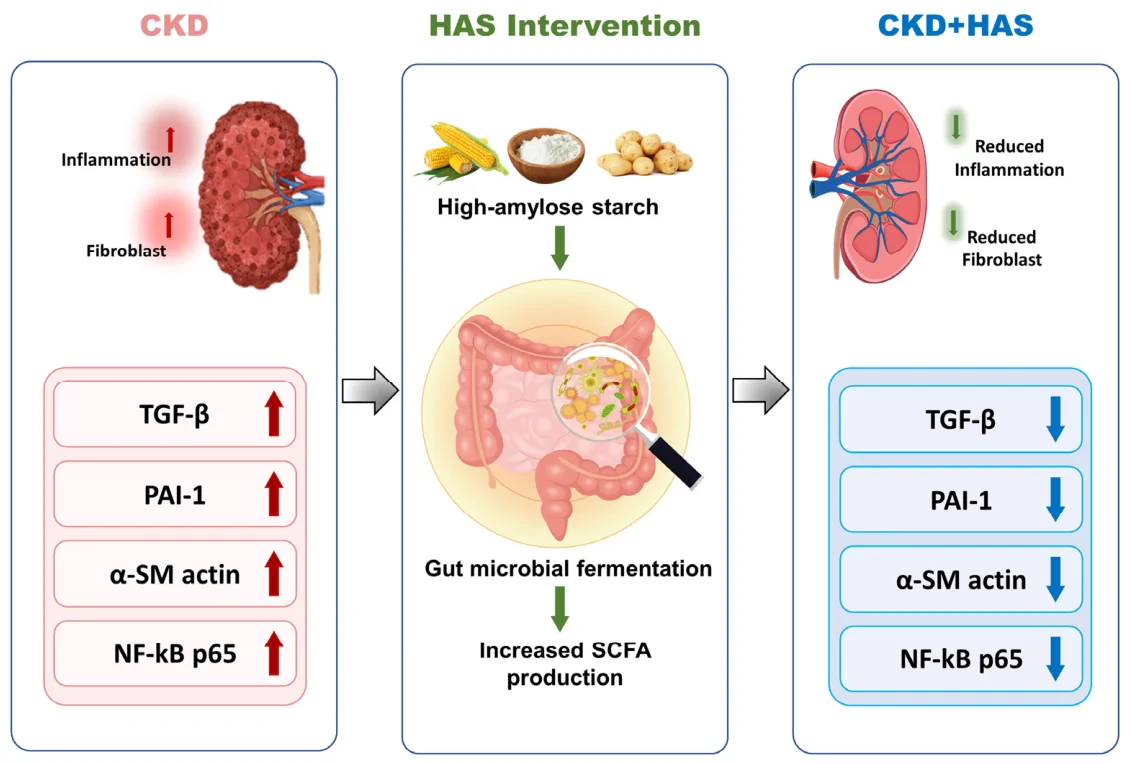
The effects of high-amylose starch on target proteins associated with chronic kidney disease (CKD). Adapted from Reference [92] under the terms of the Creative Commons Attribution 4.0 International (CC BY 4.0) license. Upward and downward arrows indicate increased and decreased expression or biological activity, respectively, whereas horizontal arrows indicate the proposed progression from CKD to HAS intervention and the resulting effects. CKD, chronic kidney disease; TGF-β, transforming growth factor beta; PAI-1, plasminogen activator inhibitor-1; α-SM, alpha-smooth muscle; NF-κB, nuclear factor kappa B; HAS, high-amylose starch; SCFAs, short-chain fatty acids.

Inflammation, gut microbiota dysbiosis, and disruption of the intestinal barrier are also associated with CKD. Among many, stimulation of some cofactors like TGF-β, PAI-1, and α-SM actin activates the fibrotic pathway in CKD [90]. On top of that, the dysfunction of the Nrf2 signaling pathway and its role in exacerbating the existing oxidative stress and inflammatory responses in CKD are noteworthy [91]. In an in vivo rodent model of CKD, male Sprague-Dawley rats were fed chow containing 0.7% adenine for 2 weeks to induce chronic interstitial nephropathy and were then randomized for 3 weeks to either a low-fiber amylopectin control diet (*n* = 9) or a high-fiber diet containing 59% high-amylose maize resistant starch type 2 (HAM-RS2; Hi-Maize 260; *n* = 9). Compared with CKD rats fed the low-fiber diet, CKD rats fed HAM-RS2 showed improved renal function and reduced renal inflammation, oxidative stress, fibrotic signaling, and tubular injury, indicating a protective effect of high-amylose resistant starch in this animal CKD model rather than direct clinical evidence in human CKD patients [92]. Consumption of the maize resistant starch diet significantly deactivated fibrotic pathway-related proteins (TGFβ, PAI-1, and α-SM actin), and partially improved the nuclear translocation of Nrf2 [92] (Figure 1). Although HAS has demonstrated therapeutic potential against CKD, current studies are limited. Further detailed investigations are needed to validate these findings and elucidate underlying mechanisms.

### 5.3. Obesity

**Figure 2:**
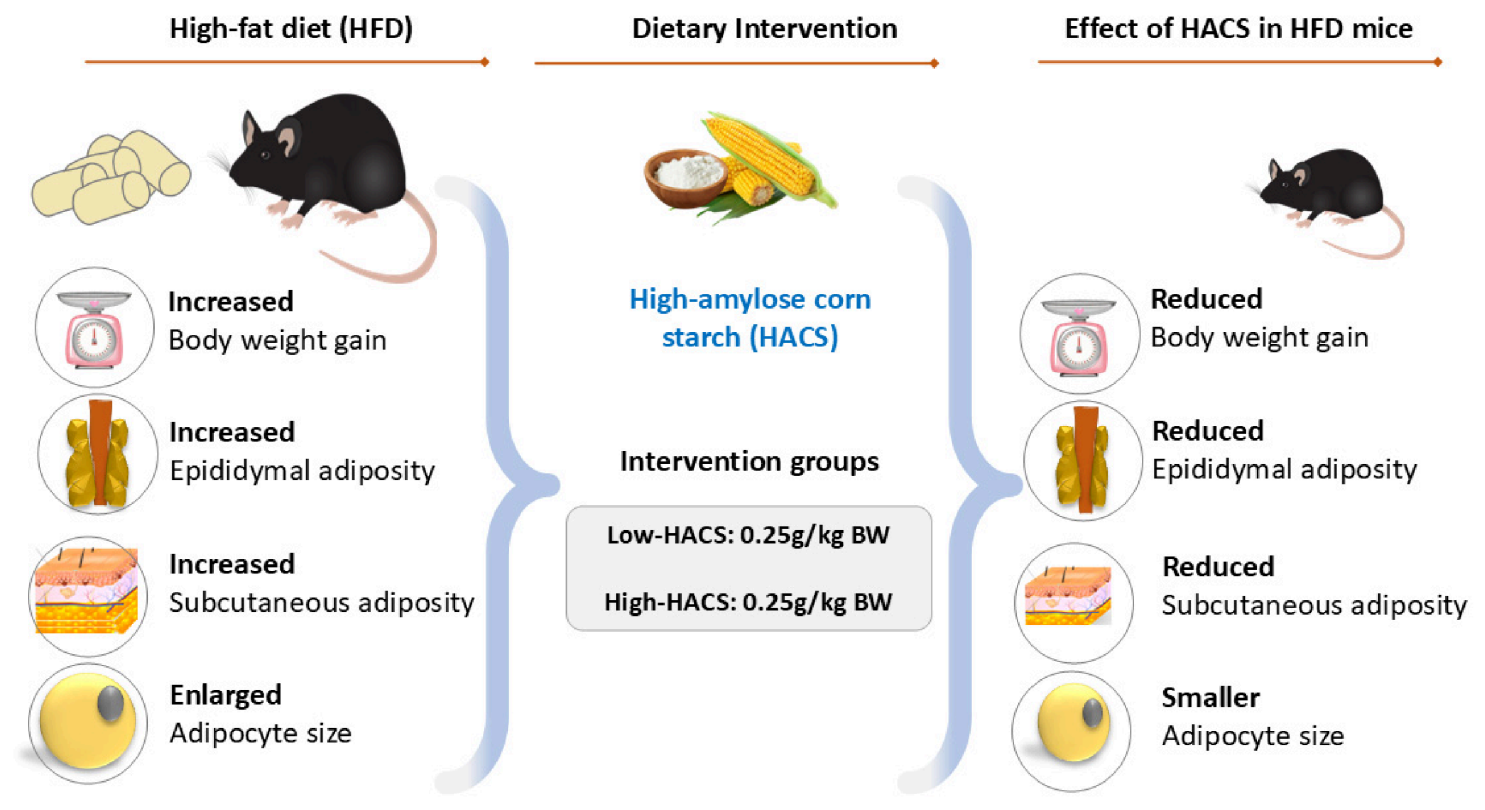
The effects of high-amylose cornstarch on body weight, adiposity, and adipocyte morphology. Adapted from Reference [93] under the terms of the MDPI open-access license, which permits unrestricted use, distribution, and reproduction provided the original work is properly cited. The figure was prepared by cropping, combining, and relabeling content from the original Figure 1 and Figure 2. HFD, high-fat diet; HACS, high-amylose cornstarch.

Obesity is a significant health concern, as it is associated with numerous health complications. In a randomized, placebo-controlled, double-blind crossover human trial involving 37 adults with overweight or obesity, participants consumed maize-derived RS2 (HAM-RS2; Hi-Maize 260, 40 g/day) or an energy-matched control starch for 8 weeks each, separated by a 4-week washout period. HAM-RS2 reduced body weight by an average of 2.8 kg and improved insulin resistance, with parallel remodeling of the gut microbiota, including enrichment of *Bifidobacterium adolescentis*. Complementary mouse experiments supported a mechanistic role for *B. adolescentis*, although broader confirmation in larger and more diverse human cohorts is still needed [73]. In a 7-week in vivo study using male ICR mice with high-fat diet-induced obesity, animals were assigned to normal diet, high-fat diet, inulin positive control, low-dose HACS (0.25 g/kg/day), or high-dose HACS (0.5 g/kg/day) groups (*n* = 5/group). HACS contained approximately 72% amylose and was administered by intragastric gavage. Compared with the high-fat diet group, HACS reduced body weight gain, adipose tissue weight, and adipocyte hypertrophy, and these changes were accompanied by altered gut microbiota composition and partial restoration of circulating bile acid profiles (Figure 2) [93]. Because this was a small preclinical mouse study, the findings support mechanistic anti-obesity potential rather than direct human efficacy [93]. Another 8-week in vivo mouse feeding study using a high-fat diet model, heat-treated brown rice starch from Dodamssal, a high-amylose rice cultivar containing 47.5 ± 0.3% amylose and 14.7 ± 1.0% resistant starch, was compared with heat-treated Samgwang medium-amylose rice starch. The high-amylose Dodamssal treatment improved glucose-related outcomes, increased fecal SCFA production, and altered gut microbiota composition, including enrichment of *Ruminococcus bromii*. However, these findings represent preclinical mouse evidence and should not be interpreted as direct confirmation of similar endocrine or microbiota effects in humans [94].

In a double-blind, randomized crossover human intervention, 33 efficacy-evaluable adults with abdominal obesity received 0 g/day control starch, 15 g/day HAM-RS2, or 30 g/day HAM-RS2 in random order for 4-week periods separated by 3-week washouts. HAM-RS2 increased insulin sensitivity in overweight and obese men, whereas the response was not equivalent across sexes, indicating that sex and baseline metabolic status may influence the glycemic response to high-amylose maize resistant starch [95]. In an acute, single-blind, randomized crossover study involving 20 overweight adults, consumption of high-amylose wheat bread at breakfast reduced postprandial glycemic responses and lowered the insulin response to a subsequent meal, with increased plasma propionate suggesting a possible fermentation-related mechanism [96]. Preclinical studies further support the metabolic effects of high-amylose starch, although these findings should be interpreted as animal evidence rather than direct clinical proof. In an 8-week in vivo study using C57BL/6J mice fed a high-fat diet, propionylated high-amylose maize starch reduced body weight gain and insulin resistance, increased fecal propionate production, and altered gut microbiota composition compared with high-fat diet controls [85]. Similarly, in a diet-induced obesity rat model, male Sprague-Dawley rats were assigned to high-amylopectin or high-amylose starch diets under ad libitum or energy-restricted feeding for 4 weeks. The HAS diet reduced energy intake, body weight gain, fat-pad mass, and glycemic response while increasing insulin sensitivity index only under ad libitum feeding, indicating that the metabolic effect depended on feeding condition [97]. Overall, evidence from small human interventions and rodent models suggests that HAS may improve insulin sensitivity, postprandial metabolism, and obesity-related outcomes; however, the magnitude of benefit appears to depend on study population, sex, starch source, dose, food matrix, and feeding context.

### 5.4. Type 2 Diabetes

HAS products may aid in the dietary prevention strategy of type 2 diabetes related to obesity [96]. HAS is more resistant to enzymatic digestion in the small intestine compared to regular starches. In the colon, HAS is fermented by the microbiome, producing SCFAs, mainly butyrate, propionate, and acetate [98]. G-protein-coupled receptor 41 (GPR41) and G-protein-coupled receptor (GPR43), located on enteroendocrine L-cells in the gut, can be activated by SCFAs [99]. Activation of these receptors by SCFAs triggers GLP-1 (Glucagon-Like Peptide-1) secretion, which plays a critical role in stimulating insulin secretion, inhibiting glucagon release, and slowing gastric emptying [100]. In a single-blind, randomized crossover human intervention, 17 adults with well-controlled type 2 diabetes consumed either 40 g/day high-amylose maize resistant starch type 2 (HAM-RS2) or a placebo for 12 weeks, separated by a 12-week washout period. Although HAM-RS2 did not improve hepatic or peripheral insulin sensitivity or HbA1c, it reduced postprandial glucose concentrations during a meal tolerance test and produced a significantly greater postprandial GLP-1 excursion than placebo (*p* = 0.009). These findings suggest that HAM-RS2 may improve meal glucose handling partly through incretin-related responses, but the evidence is limited to a small human crossover study in well-controlled type 2 diabetes [101]. With such a mechanism in place, several studies have shown that high-amylose starch reduces glycemic variability, an important factor in diabetes management. For instance, in a single-blind clinical trial involving 11 hospitalized patients with type 1 or type 2 diabetes, postprandial glucose responses were measured by continuous glucose monitoring while participants consumed control rice for 2 days and high-amylose rice “Hoshinishiki” for 2 days. Compared with control rice, high-amylose rice significantly reduced 24 h mean glucose levels, glucose levels at 2 and 3 h after meals, and postprandial glucose peak levels within 3 h, while increasing time in range. These findings suggest that high-amylose rice may improve short-term glycemic control compared with regular rice, although the evidence is limited by the small sample size, hospitalized setting, and short intervention duration [102]. In an in vivo oral rice tolerance test using six male Zucker diabetic fatty rats, high-amylose rice starch slurry was compared with common rice starch slurry. The high-amylose rice contained substantially higher apparent amylose and resistant starch contents than wild-type/common rice, and rats receiving the high-amylose rice starch slurry showed lower plasma glucose levels at 30, 60, and 90 min after administration. These findings suggest that high-amylose rice starch can attenuate acute postprandial glucose responses in a diabetic rat model, but the evidence should be interpreted as short-term preclinical data rather than direct evidence of glycemic benefit in humans [18]. Similarly, in an in vivo study using male diabetic BKS-db/db mice and non-diabetic BKS-db/m controls, animals were fed normal maize starch or one of two high-amylose resistant starch diets, including Hi-Maize 260 and NF-CGK50 for 7 weeks. HAS improved blood glucose control and attenuated diabetes-associated fragility of hepatic glycogen α-particles in db/db mice, suggesting a potential link between resistant starch intake and hepatic glucose-buffering capacity. These findings represent preclinical mouse evidence focused on glycogen structure rather than direct evidence of postprandial glucose lowering in humans [103]. HAS-modulated gut microbiome-driven SCFAs not only improve insulin sensitivity and glucose metabolism but also increase the lipid-oxidation capacity of skeletal muscle by improving mitochondrial function [104].

Clinical trials have been conducted by academic and scientific research organizations to evaluate how HI-MAIZE high-amylose maize resistant starch affects insulin sensitivity and other metabolic biomarkers. Based on those studies, and according to Ingredion, the FDA approved a qualified health claim petition for high-amylose maize resistant starch, which reduced the risk of type 2 diabetes (https://www.ingredion.com/na/en-us/news-events/news/fda-approves-qualified-health-claim-petition-for-high-amylose-ma (accessed on 10 August 2026)) [61]. They ensured that the claim was appropriately worded so as not to be misleading: “High-amylose maize resistant starch may reduce the risk of type 2 diabetes. FDA has concluded that there is limited scientific evidence for this claim.”

### 5.5. Colorectal Cancer (CRC)

Colorectal cancer (CRC) remains a major global health challenge, with increasing disease burden and substantial mortality worldwide [105]. Among human studies, the CAPP2 trial provides the strongest clinical evidence evaluating resistant starch for cancer prevention in individuals with Lynch syndrome. In this large international, randomized, placebo-controlled trial, participants with Lynch syndrome received 30 g/day resistant starch, provided as a high-amylose maize starch-derived blend of RS2 and RS3, or placebo for up to 4 years, with long-term follow-up for cancer outcomes [106]. The planned 10-year follow-up showed that resistant starch supplementation reduced non-colorectal Lynch syndrome cancers, particularly upper gastrointestinal cancers, but did not reduce colorectal cancer incidence specifically [106]. But there are human randomized biomarker studies that support a possible protective mechanism. For example, 23 healthy adults consumed 300 g/day cooked red meat with or without 40 g/day butyrylated high-amylose maize starch; HAMSB prevented the red-meat-induced increase in rectal O6MeG DNA adducts, a CRC-related biomarker. But this is not a CRC incidence trial [107].

In contrast to the CAPP2 clinical endpoint data, several animal and biomarker studies suggest that high-amylose starch may influence CRC-related mechanisms. In a 4-week preclinical rat feeding study using a high-protein meat diet model, rats were assigned to a high-protein meat control diet or diets in which part of the diet was supplemented with 10% high-amylose maize starch, high-amylose potato starch, or butyrylated high-amylose maize starch (*n* = 10/group) [108]. In this model, high-amylose potato starch and butyrylated high-amylose maize starch reduced expression of selected oncogenic miR-17-92 cluster miRNAs in the distal colon, suggesting modulation of cancer-related molecular markers [108]. The miR-17-92 cluster is frequently associated with oncogenic transformation and tumor progression [109]. However, the same study did not show significant diet-induced differences in colonic O6-methyl-2-deoxyguanosine (O6MeG) DNA-adduct levels, indicating that the biomarker response was not uniform across all CRC-related endpoints [108]. Therefore, these findings support a possible mechanistic effect of selected high-amylose starches on colonic molecular markers, but they should not be interpreted as direct evidence of CRC prevention in humans.

Additional preclinical evidence comes from azoxymethane-induced intestinal cancer models. In one in vivo rat study, four groups of Sprague-Dawley rats (*n* = 30/group) were fed AIN-93G-based diets containing standard low-resistant-starch maize starch, low-resistant-starch maize starch plus tributyrin, 10% high-amylose maize starch, or 10% butyrylated high-amylose maize starch [110]. Compared with the low-resistant-starch control diet, both high-amylose maize starch and butyrylated high-amylose maize starch reduced the proportion of rats developing tumors, while butyrylated high-amylose maize starch also reduced tumor number per rat [110]. These results suggest that high-amylose maize starch, particularly when chemically modified to deliver butyrate, can suppress tumor development in a carcinogen-induced rat model. However, this remains preclinical evidence using chemically induced tumorigenesis and should be distinguished from clinical CRC outcomes in humans.

A separate azoxymethane-induced rat study further reported that diets containing 10% or 20% high-amylose maize starch reduced CRC incidence and increased apoptosis in distal colonic crypts compared with a control diet lacking dietary fiber or resistant starch [111]. These findings suggest that high-amylose maize starch may enhance removal of DNA-damaged or abnormal epithelial cells in experimental carcinogenesis. Similarly, in a rat dietary model comparing beef- and chicken-containing diets with or without high-amylose maize starch, high-amylose maize starch altered circulating CRC-related markers, including interleukins and matrix metalloproteinase-related factors [112]. These data provide additional mechanistic support, but the outcomes were circulating or tissue biomarkers rather than clinical CRC incidence.

The possible role of microbial metabolites also supports a biologically plausible link between resistant starch fermentation and tumor-related immune regulation. Short-chain fatty acids (SCFAs), especially butyrate, are major fermentation products of resistant starch and have been implicated in epithelial homeostasis, immune regulation, and antitumor responses. In experimental cancer immunology models, SCFAs such as pentanoate and butyrate enhanced cytotoxic T-cell and CAR-T-cell antitumor activity through metabolic and epigenetic reprogramming [113]. However, this study was not a dietary HAS intervention and was not specific to CRC prevention; therefore, it should be used only as mechanistic support for SCFA-mediated immune modulation.

Evidence from other cereal polysaccharides also suggests that fermentable carbohydrate structures may influence CRC-related pathways. For example, in an AOM/DSS-induced colorectal cancer mouse model, barley polysaccharide gavage at 300 mg/kg/day reduced tumor number and volume, preserved colon length, and decreased inflammatory cytokines, including IL-1β, IL-4, and IL-17A [114]. However, because this intervention involved barley polysaccharides rather than high-amylose starch specifically, it should be interpreted as related evidence for cereal-derived fermentable carbohydrates rather than direct HAS evidence.

**Figure 3:**
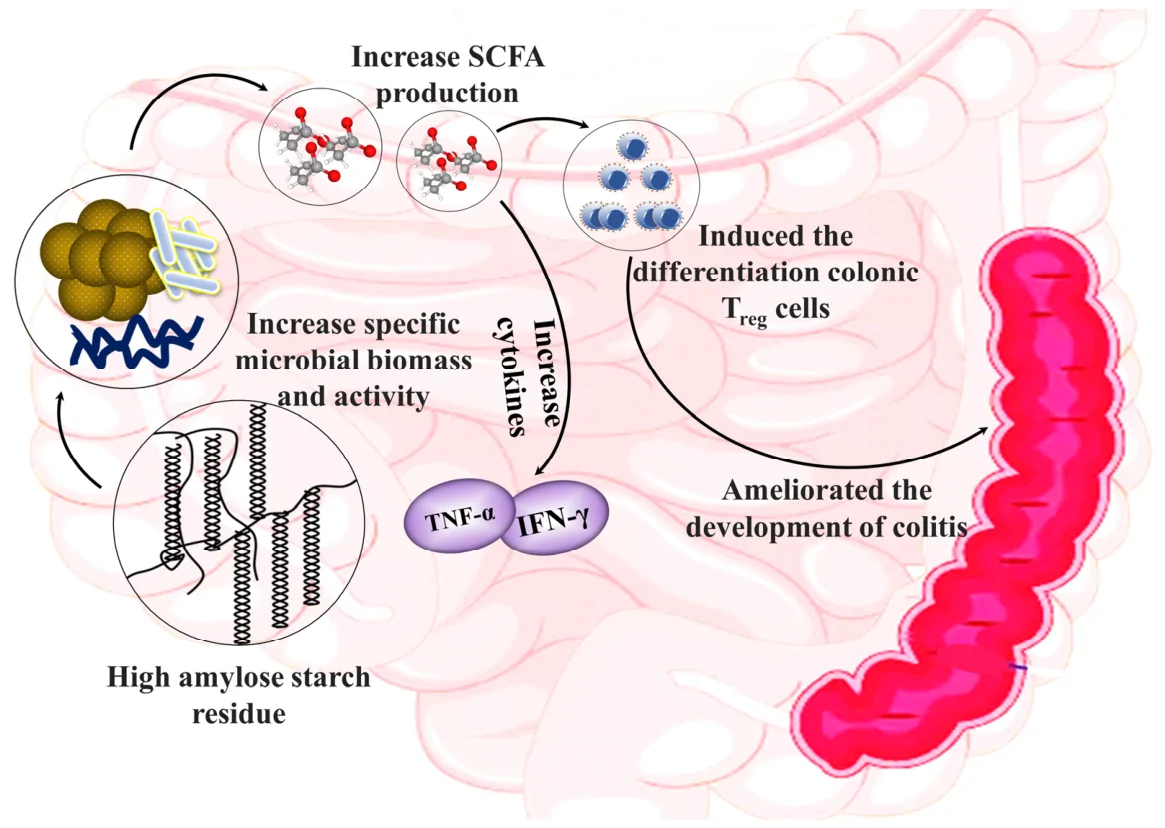
Potential modulation of colitis- and CRC-related inflammatory pathways by high-amylose starch through gut microbiota-derived short-chain fatty acids (SCFAs). The figure summarizes potential effects on fermentation, butyrate production, epithelial apoptosis, inflammatory signaling, and antitumor immune responses. SCFA, short-chain fatty acid; TNF-α, tumor necrosis factor alpha; IFN-γ, interferon gamma; Treg, regulatory T cell; CTL, cytotoxic T lymphocyte. This figure was created based on references [39,107,110,111,113].

Taken together, the CRC-related literature indicates a clear difference between preclinical mechanistic findings and human clinical endpoint evidence. Rodent and biomarker studies suggest that high-amylose starch and related resistant starch preparations may alter fermentation, SCFA production, oncogenic miRNA expression, epithelial apoptosis, inflammatory signaling, and tumor development in experimental models. However, the CAPP2 randomized trial did not show reduced colorectal cancer incidence in Lynch syndrome patients. This discrepancy may reflect differences in cancer etiology, including hereditary Lynch syndrome versus chemically induced or sporadic-like animal models; differences in dose, starch type, food matrix, intervention timing, and duration; interindividual differences in gut microbiota responsiveness; or a gap between biomarker modulation and hard clinical endpoints. Therefore, HAS should be described as a promising dietary component that may modulate CRC-related biological pathways (Figure 3), but current evidence is insufficient to conclude that HAS prevents CRC in humans.

## 6. Metabolic Alteration by High-Amylose Starches

The source of HAS (e.g., corn, barley, wheat, potato) influences its structure, fermentation properties, and subsequent metabolic effects [8,115,116]. HAS from different botanical sources has demonstrated distinct metabolite alteration profiles across diverse biological models, driven by variations in structure, fermentability, and interactions with the gut microbiota (Table 6). Corn-derived HAS significantly influences lipid metabolism, as evidenced by reduced plasma cholesterol, increased bile acid pool size, suppressed hepatic HMG-CoA reductase expression, and elevated cecal propionate levels in rats [117]. In mice, it upregulates a broad spectrum of metabolites, including amino acids (tyrosine, methionine), SCFAs, bile acids, and redox cofactors (NAD^+^, riboflavin), reflecting enhanced microbial fermentation and systemic metabolic shifts [118]. In contrast, high-amylose barley and wheat, when integrated into human diets, consistently elevate fecal butyrate and propionate concentrations while reducing p-cresol, a uremic toxin, suggesting favorable microbiota-mediated fermentation and reduced colonic toxicity [96,119]. Rice with elevated amylose content, in vitro and in rats, boosts SCFAs (butyrate, acetate, propionate) and increases plasma potassium, further affirming its prebiotic potential [120]. Complementing these findings, high-amylose pea starch in pigs enhanced liver fatty acid β-oxidation. It increased the levels of unsaturated fatty acids and amino acids, suggesting systemic metabolic remodeling beyond the gut [18]. In a randomized crossover human feeding study involving 23 hypertriglyceridemic, mostly abdominally obese adults, consumption of a HAS diet for 4 weeks increased fecal–water SCFA concentrations by 32% and modestly reduced postprandial insulin response during a test meal, but did not significantly change fasting glucose or insulin. Therefore, this study supports an effect of HAS on colonic fermentation and postprandial insulin handling, but not direct evidence of improved insulin sensitivity [121]. Collectively, these findings highlight the metabolite-modulating effects of HAS, emphasizing source-specific and host-dependent outcomes that could inform functional food design and metabolic disease interventions.

In human diets, HAS is usually consumed in processed or composite foods such as bread, rice, noodles, pasta, steamed buns, or baked products rather than as purified native starch. Processing methods such as cooking, cooling, baking, steaming, and extrusion can alter starch structure, granule integrity, and amylose–lipid complex formation, thereby modifying enzymatic digestibility and the amount of resistant substrate reaching the colon [36,133]. Processing conditions that preserve native granular structure, promote retrogradation after cooking, or favor amylose–lipid complex formation may enhance enzymatic resistance, whereas extensive cooking, severe shear, or extrusion can disrupt starch structure and increase digestibility. Therefore, the physiological effects and health claims of HAS-based foods should be interpreted based on the final processed food matrix rather than amylose content alone.

## 7. Gut–Brain Axis

**Figure 4:**
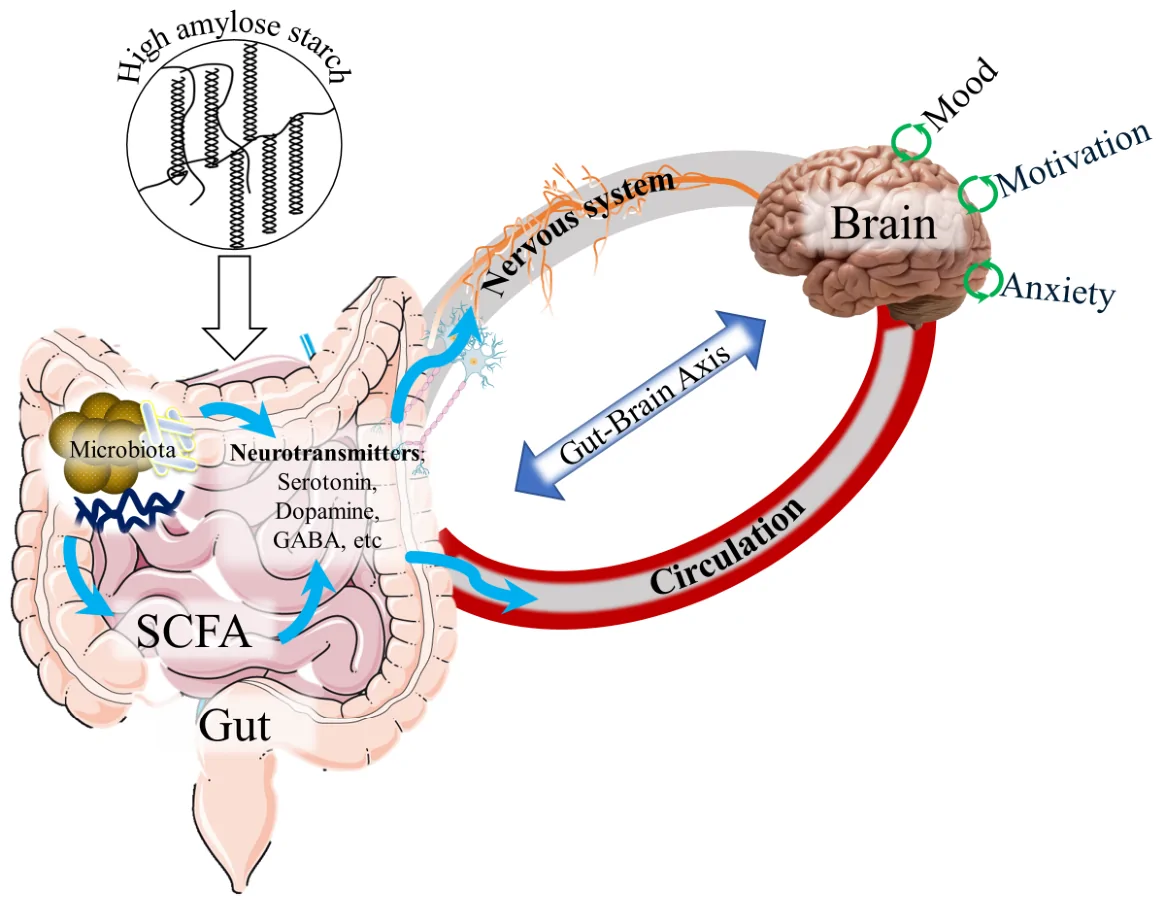
Potential mechanisms by which high-amylose starch modulates the gut–brain axis. High-amylose starch may influence gut–brain communication by reshaping the gut microbial community and increasing the production of short-chain fatty acids, thereby modulating neuroimmune signaling, metabolic homeostasis, and bidirectional communication between the gastrointestinal tract and the central nervous system. SCFA, short-chain fatty acid; GABA, gamma-aminobutyric acid.

The gut–brain axis (GBA) encompasses a bidirectional communication system that facilitates interaction between the central nervous system and the enteric nervous system, thereby linking emotional and cognitive domains of the brain with peripheral gastrointestinal functionalities [134]. The GBA is a bidirectional communication network between the central nervous system (brain) and the gut, influenced by the microbiome. Neurotransmitters, like serotonin, dopamine, and GABA, act as chemical messengers that travel between the gut and brain, influencing various functions, including mood, digestion, and stress response [135]. HAS can contribute to those neurotransmitters by altering the microbial community and producing SCFAs (Figure 4).

Gamma-aminobutyric acid (GABA) serves a fundamental function within the central nervous system as a neurotransmitter that exerts inhibitory effects. Low levels of GABA, a neurotransmitter that inhibits nerve cell activity, can contribute to various neurological and mental health problems, including depression, anxiety, and seizure disorders [136]. Several gut microbes, including Bacteroides, Lactobacillus, and Bifidobacterium, can produce or modulate GABA levels in the gut, impacting the gut–brain axis [137]. In a 21-day randomized study using 32 weaned pigs, diets containing progressively higher amylose levels increased hindgut fermentation and promoted *Bifidobacterium* spp. abundance, particularly with the 63% amylose diet [72]. A 4-week study in 32 male Sprague-Dawley rats similarly showed that a HAS-containing diet increased cecal _Bifidobacterium_ numbers and SCFA pools compared with low-amylose starch diets, although the response was modified by deoxycholic acid exposure [138].

In a 28-day weanling pig study, diets containing 40% high-amylose cornstarch increased cecal *Lactobacillus* and *Terrisporobacter* and decreased *Streptococcus* abundance, based on cecal digesta collected from one pig per pen (*n* = 8/treatment) [81]. In a 35-day randomized feeding study using 27 Xiangdong black female goats, the diet containing 50% high-amylose corn enriched *Lactobacillus* and *Faecalibacterium* in cecal digesta and *Akkermansia* in mucosa compared with the 0% amylose corn diet [66]. In vitro fermentation using fecal inoculum from 17 healthy infants further showed that high-amylose maize starch and acetylated high-amylose maize starch were fermented by infant fecal microbiota, with weaning-stage inoculum showing increased microbial diversity and higher *Bifidobacterium* and *Bacteroides* abundance [139]. Overall, in the gut, the presence of SCFAs can create a favorable environment for GABA-producing bacterial growth by maintaining gut barrier integrity and modulating immune responses, thereby indirectly supporting increased GABA production [140,141].

Serotonin plays a crucial role in various cognitive functions that are vital for interacting within the social sphere, encompassing emotional regulation and impulse management [142]. Microbiota such as *Streptococcus* spp., *Enterococcus* spp., *Escherichia* spp., *Lactobacillus plantarum*, *Klebsiella pneumonia*, and *Morganella morganii* can produce serotonin [143]. Although evidence did not find any alteration in serotonin-producing bacteria by a high-amylose diet. But studies suggested that Gut microbial metabolites SCFAs, stimulate enteric serotonin synthesis and are responsible for maintaining gut health [144]. In an in vitro Caco-2/TC7 intestinal epithelial cell study, 24 h exposure to SCFAs showed that butyrate significantly increased serotonin transporter (SERT) expression at both the mRNA and protein levels. These findings suggest that microbial SCFAs may regulate intestinal serotonin signaling, but they provide mechanistic cell-culture evidence rather than direct evidence from HAS feeding or in vivo gut–brain axis studies [145]. Another neurotransmitter, dopamine (DA), regulates reward and movement in the brain. DA is primarily secreted in the ventral tegmental area and the substantia nigra within the brain, but some gut microbiota can directly produce dopamine, and some can influence dopamine levels through enzymatic influence [146]. A recent review summarized preclinical evidence that SCFAs may modulate dopaminergic signaling by influencing tyrosine hydroxylase expression and NF-κB-related oxidative and inflammatory pathways. Because this evidence is not from HAS feeding studies, it should be interpreted as indirect mechanistic support for SCFA-mediated gut–brain axis effects rather than direct evidence that HAS increases brain dopamine levels [147]. SCFA treatment increased dopamine receptor D1a (DRD1a) expression, independent of psychosocial stress, whereas dopamine receptor D2 (DRD2) gene expression remained unaffected. However, several studies reported that many bacteria can produce dopamine in the gut, including bacilli, *E. coli*, *Proteus vulgaris*, *Serratia marcescens*, *Staphylococcus aureus*, *Hafnia alvei*, and *Klebsiella pneumoniae* [148]. HAS can influence some of those microbes, but no direct study has investigated the effect of high amylose on gut dopamine. Further study is needed on the role of HAS in modulating the dopamine-producing gut microbiota. Existing findings indicate that HAS can indirectly influence dopamine levels [141]. Several other neurotransmitters are also produced by gut microbiota, including norepinephrine, acetylcholine, and histamine. There is significant potential in the study of HAS’s role in modulating the production of these neurotransmitters.

## 8. Future Studies and Limitations of This Study

High-amylose starch has clear potential as a microbiome-active carbohydrate, but the next wave of research should move from “does it work?” to “for whom, how, and under what conditions does it work best?” A major gap is response variability: because baseline gut microbiome composition differs widely, some individuals show strong metabolic and microbial benefits from HAS. In contrast, others respond weakly or not at all. This makes a compelling case for precision nutrition, in which HAS recommendations are guided by microbial features (e.g., fermentative capacity, key taxa, or functional gene profiles) rather than by a one-size-fits-all dose. Future studies could include stratified randomized controlled trials in which participants will be grouped according to baseline resistant-starch fermentation capacity. For example, participants enriched in starch-degrading taxa such as *Ruminococcus bromii* and *Bifidobacterium adolescentis*, together with amylolytic or cross-feeding taxa such as *Bacteroides thetaiotaomicron*, *Bacteroides ovatus*, *Eubacterium rectale*, and *Roseburia* spp., could be compared with low-fermentation responders after defined HAS interventions. Parallel measurements of microbiota composition, SCFA production, glycemic response, and inflammatory markers would help determine whether baseline microbial capacity predicts HAS responsiveness. While acetate, propionate, and butyrate are central, HAS likely reshapes broader metabolite networks, including clinically relevant molecules such as choline chloride, trimethylamine-N-oxide (TMAO), and bile acid derivatives, through microbiota-driven pathways that remain only partially mapped. Equally important is clarifying the mechanisms by which resistant starch type, different HAS sources, and resistant starch forms can deliver distinct structures to the colon, potentially activating distinct microbial enzymes and metabolic pathways.

To address these gaps, future studies should integrate shotgun metagenomics, metabolomics, and host transcriptomic analyses. This multi-omics approach will help identify the microbial genes, fermentation metabolites, and host response pathways through which high-amylose-derived resistant starch influences gut health, glycemic regulation, and inflammation. Studies should emphasize functional validation, linking shifts in taxa to pathway activity and metabolite outputs, not just changes in relative abundance. Finally, the field needs well-powered, long-term clinical trials that evaluate sustained effects on insulin sensitivity, lipid profiles, inflammatory markers, and gut barrier function, while tracking adherence and real-world feasibility. Trials in high-need populations—such as individuals with chronic kidney disease, metabolic syndrome, or colorectal cancer risk—could reveal where HAS has the strongest therapeutic relevance. There is also room for discovery by exploring underutilized botanical sources of HAS and by testing synergies with other fibers, polyphenols, and probiotics/symbiotics that may amplify benefits. Together, these priorities can transform HAS from a promising functional ingredient into a targeted, evidence-based tool for improving gut ecology and metabolic health.

According to Ingredion Incorporation (https://www.ingredion.com/apac/en-sg/ingredients/ingredient-product-families/hi-maize-resistant-starch (accessed on 10 August 2026)), there are more than 80 published clinical studies that demonstrate the metabolic and physiological benefits of natural HI-MAIZE high-amylose resistant starch, particularly in optimizing gastrointestinal health, facilitating weight management, and regulating glycemic and energy responses. In this review, some of the evidence is based on animal and in vitro studies. These findings should therefore be interpreted cautiously and not as direct clinical evidence. Nevertheless, animal and experimental studies provide useful mechanistic insight and can serve as a foundation for designing future human trials to evaluate the effects of HAS on gut microbiota, microbial metabolites, metabolic health, and disease prevention.

We have focused on high-amylose starch in this review. We should note that high-amylose wheat and its flour are now commercially available. High-amylose wheat flour can be directly formulated to make wheat-based foods and has great potential to offer significant health benefits due to its high-amylose starch content.

## 9. Conclusions

HAS is a promising microbiome-active carbohydrate that may support gut and systemic health by resisting digestion in the upper gastrointestinal tract and providing a fermentable substrate for colonic microbiota. Human clinical studies have demonstrated the metabolic and physiological benefits of HAS. Current evidence indicates that HAS can alter gut microbial composition, increase SCFA production, and influence pathways related to glucose homeostasis, lipid metabolism, adiposity, inflammation, and gut barrier function. However, many reported effects remain associative or are based on animal, in vitro, or biomarker studies; therefore, HAS-induced microbiota changes should be interpreted as plausible mediators rather than definitive proof of causation. In addition, HAS should be viewed as a functional category rather than a uniform ingredient because its effects depend on botanical source, starch structure, processing history, and food matrix. Overall, HAS holds potential as a prebiotic and metabolic modulator, but disease-specific benefits, particularly for CKD, CRC, and gut-brain axis outcomes, require further investigation using well-powered human trials and functional microbiota validation studies.

## Figures and Tables

**Table 1:** Major sources of high-amylose starch and technologies used to enhance amylose content.

Crop Source	Technology/Strategy to Increase Amylose	Amylose Content * (%) Natural (Max)	Amylose Content (%) Enhanced * (Max)	Reference
Experimental Samples				
Maize	CRISPR/Cas9-mediated editing of starch branching enzyme IIb (SBEIIb; Zm00001d016684).	31.63	53.48	[10]
Maize	Marker-assisted backcross breeding.	26.39	58.10	[11]
Maize	U6 promoter and U6 terminators were cloned into HindIII and PstI site with the maize codon-optimized Cas9 gene.	19.31	40.06	[12]
Maize	Reduced expression of starch branching enzyme IIa and IIb	29.07	55.89	[13]
Maize	Introgressed the Ae1-5180 gene into the Ae1-5180 mutant maize.	26.08	47.23	[14]
Maize	Guatemalan breeding cross	-	85.6	[15]
Maize	Multiple mutation (du su variety)	29	70	[16]
Rice	Antisense RNA inhibition of starch branching enzymes	29.98	58.32	[17]
Rice	Inhibition of two isoforms of starch branching enzyme (SBE), SBEI and SBEIIb	27.2	64.8	[18]
Rice	Wx Transgene into Rice wx Mutants Leads	-	46.3	[19]
Wheat	Transgenic modification of starch biosynthesis genes.	25.00	55.00	[20]
Wheat	Durum wheat near-isogenic lines.	45.10	69.80	[21]
Wheat	Triple SGP-1 mutant line crossed with ‘Ukrainka’, ‘Lona’, and ‘Solstice’.	24.85	43.03	[22]
Wheat	Transgenic wheat: suppression of SBEIIa and SBEIIb expression.	31.80	88.50	[23]
Wheat	Knock-out mutations in starch synthase IIa (SSIIa) across the three genomes of wheat cultivar ‘Jagger’.	31.15	35.70	[24]
Wheat	Mutagenized durum wheat line (DHA175).	38.00	53.60	[25]
Potato	Antisense-mediated suppression of starch branching enzyme SBEI and SBEII (retransformation approach).	25.59	89.14	[26]
Potato	Solanum sandemanii introgression followed by repeated backcrossing to *Solanum tuberosum* lines.	21.00	37.90	[27]
Potato	Inhibition of SBE1 and SBE2 genes encoding starch branching enzymes.	20.00	87.00	[28]
Potato	DNA-free genome editing to induce branching enzyme genes.	25	98	[29]
Barley	Genome editing to alter starch branching.	12.28	73.61	[30]
Barley	Mutagenized using sodium azide.	25.0	71.7	[31]
Barley	Using a chimeric RNAi hairpin suppressed SBE I, SBE IIa, SBE IIb.	29	65	[32]
Barley	Provided by PlantCarb ApS, Hørsholm, Denmark		82.7–99.8	[33]
Commercial Samples				
Maize	HYLON V starch was developed by selective plant breeding and is available commercially from Ingredion.	-	55.0	[34]
Maize	HYLON VII starch was developed by selective plant breeding and is available commercially from Ingredion	-	73.8	[34]
Maize	Gelose 50 starch is commercially available from Penford Australia, now part of Ingredion.	-	50.0–59.0	[35]
Maize	Gelose 80 starch is commercially available from Penford Australia, now part of Ingredion.	-	80.0	[35]
Maize	Amylogel™ 03001, a high-amylose maize starch, was developed by selective breeding and is available commercially from Cargill.	-	59.1	[36]
Maize	Amylogel™ 03003, a high-amylose maize starch, was developed by breeding and is available commercially from Cargill.	-	68.2	[36]
Maize	AMYLO STARCH N-400 was developed by selective breeding and is available commercially from Roquette.	-	65.0	[37]
Pea	Commercial pea starch from Parrheim Foods (P&H Milling Group).	-	41.10	[38]
Pea	NASTAR pea starch is commercially available from COSUCRA	-	35.5	[39]
Pea (Ingredion)	Commercial Purity P 1002 native pea starch is commercially available from Ingredion.	-	30.44	[40]
Pea	Commercial ingredient	-	33.0	[41]
Pea	Commercial ingredient	-	40.7	[42]
Pea	Commercial ingredient available from Roquette Canada Ltd.	-	40.70	[38]

* In experimental samples, natural amylose content refers to the reported wild-type, parent, or control material, and enhanced amylose content refers to the modified or selected high-amylose sample. In commercial samples, matched natural/wild-type baselines were often not reported because the starches were presented as commercial ingredients; therefore, only the reported amylose content of the commercial product is listed, and “-“ indicates not reported or not applicable.

**Table 2:** Mechanism-based classification of high-amylose starch according to enzyme resistance pathways.

Mechanism-Based HAS Category	Primary Mechanism of Enzyme Resistance	Examples	Reference
Native granular/crystalline HAS	Intact starch granules, compact crystalline organization, and limited enzyme attack sites reduce enzymatic hydrolysis.	Native high-amylose maize, rice, wheat, barley, and potato starches.	[47]
Molecular structure-based HAS	Increased amylose content, longer amylopectin branch chains, altered branching pattern, and modified crystalline/thermal properties reduce enzyme accessibility.	SBE-inactivated, mutant, transgenic, or gene-edited high-amylose cereals and tubers.	[48]
Retrograded HAS/RS3-like structures	Gelatinized starch chains, especially amylose, reassociate during cooling or storage to form ordered structures that resist digestion.	Cooked-and-cooled HAS foods, cooled rice, bread, noodles, pasta, and steamed products.	[49]
Amylose–lipid inclusion complex HAS/RS5-like structures	Amylose forms single-helical inclusion complexes with lipids, reducing enzyme accessibility and slowing starch digestion.	HAS is processed with lipids, emulsifiers, fatty acids, or lipid-containing food matrices.	[50,51]
Chemically modified or acylated HAS	Chemical substitution or acylation reduces digestibility and may deliver specific SCFAs, such as butyrate or propionate, to the colon.	Butyrylated high-amylose maize starch, propionylated high-amylose maize starch, acetylated starches.	[52,53]

Note: These categories should be interpreted as overlapping functional categories rather than a strict taxonomy. A single HAS product may belong to more than one category simultaneously; for example, a chemically modified or acylated starch may also undergo retrogradation or form amylose–lipid complexes depending on processing conditions and food matrix.

**Table 3:** Digestion of high-amylose starch across regions of the gastrointestinal tract.

Region	Key Process	Reference
Mouth	Salivary amylase hydrolyzes amylose into oligosaccharides and dextrins.	[55]
Stomach	Limited digestion due to acidic pH; starch granules remain intact.	[56]
Small Intestine	Pancreatic amylase and brush-border enzymes break down amylose into glucose.	[8]
Colon	Resistant starch is fermented by gut microbiota, producing SCFAs.	[59]

Abbreviation: SCFAs, short-chain fatty acids.

**Table 4:** Reported effects of high-amylose starch consumption on selected beneficial gut microbiota.

Microbiota	Function	Starch Type/Source	Effect on Microbiota	Outcome	Reference
*Bifidobacteria*	Often regarded as beneficial; associated with immune development and higher neutrophils, basophils, plasmablasts, and memory CD8+ T cells.	Rice starch (0% and 20% amylose; Remyline AX-DR, Remy B7), pea starch (35.5% amylose; Nastar), and maize starch (80% amylose; Gelose).	Starch containing 63% amylose increased *Bifidobacterium* spp. compared with starches containing <5%, 20%, or 28% amylose.	Higher amylose (and lower in vitro digestibility) increased post-ileal substrate delivery and fermentation, selectively enriching *Bifidobacterium* spp. in the distal gut.	[64,72]
*Lactobacillus*	Supports intestinal barrier function and may reduce gut injury by strengthening immune, epithelial, and mucus barriers.	Commercial high-amylose cornstarch (Hainan Shanliang Technology Co., Ltd.).	A diet with 50% high-amylose starch increased Lactobacillus in the digesta and increased *Akkermansia* in the mucosa versus a 0% amylose control.	Lower fatty-acyl-related metabolites were observed, consistent with a healthier cecal microbiota profile and improved host immune function.	[65,66]
*Faecalibacteria*	Key butyrate-producing commensal; helps regulate immunity (Th17/Treg balance), reduces inflammatory cytokines, and supports gut barrier integrity.	Butyrylated high-amylose maize starch (Ingredion Incorporated; formerly National Starch and Chemical Company).	High-amylose maize starch increased *Faecalibacterium prausnitzii* abundance (~5.1-fold) compared with low-amylose maize starch.	Total butyrate increased with high-amylose starch; plasma IL-10 and TNF-α increased in one study, while other immune indices were unchanged.	[53,67]
*Ruminococcus*	Degrades complex carbohydrates (e.g., cellulose), releasing substrates that other microbes and the host can use.	Refined wheat starches with high-amylose vs. low-amylose profiles.	High-amylose wheat starch increased *Ruminococcus* spp. relative to the low-amylose wheat starch control.	High-amylose wheat starch consumption was associated with changes in fecal butyrate excretion.	[68,69,70]
*Akkermansia*	Mucin-degrading genus linked to improved barrier integrity and immune regulation.	Maize-derived high-amylose starch from ae1/sbe1 lines.	Individuals consuming high-amylose maize starch showed greater enrichment of *Akkermansia* than those consuming control starch.	In vivo, high-amylose starch improved gut barrier function, reduced chronic inflammation, and lowered blood glucose in high-fat diet (HFD) models.	[12,71]

Abbreviation: CD8+, cytotoxic differentiation 8 T cell; Th17, T helper 17 cells; IL-10, interleukin-10; TNF-α, tumor necrosis factor alpha; HFD, high-fat diet.

**Table 5:** Effect of high-amylose starch consumption on harmful gut microbiota.

Name	Function of the Microbiota	Effect of (HAS) on the Microbiota	Reference
*Escherichia coli* (pathogenic strains)	Pathogenic strains can cause urinary tract infections, diarrheal disease, and invasive infections (e.g., sepsis or meningitis).	A high-amylose starch diet (PS) reduced *Escherichia coli* abundance compared with a low-amylose diet (TS).	[74,75]
*Clostridium difficile*	*Clostridium difficile* causes nosocomial infectious diarrhea and can end in pseudomembranous colitis and toxic megacolon.	*Clostridium* clusters IV and XIVa were decreased in pigs consuming high amylose (63%) compared with low amylose (0%, 20%, and 28%).	[72,76]
*Salmonella* spp.	Foodborne zoonotic pathogens that cause gastroenteritis; some infections are associated with an increased risk of hepatobiliary and colorectal diseases.	High-amylose potato starch reduced fecal Salmonella in pigs; in human studies, HAS reduced multidrug-resistant Salmonella enterica serovar I 4,[5],12:i:-	[77,78,79]
*Streptococcus* spp.	Higher gut *Streptococcus* abundance has been associated with systemic inflammation and cardiovascular risk markers.	High-amylose cornstarch decreased the relative abundance of *Streptococcus*.	[80,81]
*Bacteroides* (selected species)	Some species contribute to reservoirs of antimicrobial resistance genes and may behave as pathobionts under dysbiosis.	In vitro, growth media containing high-amylose maize starch inhibited the growth of *Bacteroides fragilis* and *Bacteroides vulgatus*.	[82,83]
*Erysipelotrichaceae* (unclassified genera)	Higher abundance has been reported in colorectal cancer and other inflammatory states.	Relative to a high-fat diet control, high-amylose maize starch lowered the abundance of unclassified Erysipelotrichaceae.	[84,85]

Abbreviation: PS, high-amylose-starch diet; TS, low-amylose diet; HAS, high-amylose starch.

**Table 6:** Effect of high-amylose starch supplementation on metabolite production.

Source of Amylose	Percentage of Treatment	Study Model/Species	Affected Metabolites	Reference
High-amylose cornstarch/maize starch	200 g/kg diet	Sham-operated and cecectomized rats	Lowered plasma total cholesterol levels. Increased intestinal bile acid pool identified. Reduced hepatic HMG-CoA reductase mRNA in cecectomized rats. Elevated propionate concentration in the cecal contents of sham-operated rats. Enhanced biliary bile acid flux into the small intestine.	[117]
High-amylose cornstarch/maize starch	Supplemented 20% in AIN93G rodent diet	C57BL/6 male mice	The levels of tyrosine, tryptophan, kynurenate, anthranilate, xanthurenate, 5-hydroxyindoleacetate, IPA, GSH (reduced), glycine, leucine, methionine, asparagine, succinate, fumarate, malate, cholesteryl sulfate, TMAO, β-hydroxybutyrate, cholate, deoxycholate, NAD (NAD^+^), and riboflavin (vitamin B_2_) were increased in the circulation.	[118]
High-amylose cornstarch/maize starch	150 g/kg diet	Ovariectomized (OVX) rats	Plasma total cholesterol and LDL cholesterol concentrations declined. The farnesoid X receptor mRNA levels, along with fecal bile acids and neutral sterols excretion, were elevated relative to a high-sucrose diet.	[122]
High-amylose cornstarch/maize starch	40% high-amylose cornstarch	Pig	Significantly increased ceceal butyrate and volatile fatty acids by 40% high amylose, with 40% cold pressed canola cake supplementation in a common commercial diet.	[81]
High-amylose cornstarch/maize starch	21% acidified high-amylose cornstarch in a high-fat diet (45% lipid)	C57BL/6J male mice	Plasma alanine aminotransferase (ALT), LDL-C, HDL-C, and TG decreased significantly compared to the high-fat diet. Cecum stool acetic acid and Propanoic acid were found to be significantly higher, but butyric acid was found to be non-significantly higher.	[123]
High-amylose cornstarch/maize starch	9.97 mg/mL digested high-amylose cornstarch	In vitro	Compared to basal medium, the production of acetate, propionate, butyrate, and total SCFAs (D) during fermentation was significantly higher in the high-amylose cornstarch group.	[124]
High-amylose cornstarch/maize starch	40% high-amylose starch in a basal diet.	Pig	Significantly increased volatile fatty acids, Acetate, Propionate, Butyrate, and branched-chain fatty acids (*p* = 0.09) in the cecum.	[81]
High-amylose cornstarch/maize starch	0.025–25 mg/kg VB12 with and without 5–30% high-amylose cornstarch	Sprague-Dawley male rat	Only 20% of high-amylose cornstarch supplementation significantly increased cecal succinate concentration. 20–30% high-amylose cornstarch supplementation increased cecal cobalamin by 75% compared to control. Propionate decreased in cecal by 30% high amylose cornstarch by the addition of sufficient VB12 increased propionate.	[125]
High-amylose cornstarch/maize starch	30% wheat flour replaced with high-amylose maize starch in Chinese steamed buns.	Human	Significantly decreased blood glucose.	[126]
High-Amylose Barley	High-amylose barley variety flour in bread 20%, crackers 37%, and muffins 21%.	Human	Increased butyrate concentration in feces (42%) and 91% in excretion. Increased total SCFA in feces by 57% but decreased p-cresol concentration by 33%.	[119]
High-Amylose Barley	50% amylose-only barley flour (AmOn)-containing bread	Human	Plasma gastric inhibitory peptide (GIP) was reduced, but free fatty acids were increased significantly.	[127]
High-amylose wheat	Two 85% and 75% high-amylose flour-containing breads	Human	Propionate levels rose by 9% and 12% six hours post consumption of 85- and 70% high-amylose wheat breads, respectively.	[96]
High-amylose wheat	15%, 20%, and 45% amylose-containing wheat noodles	Human	Individuals who consumed noodles with a 45% amylose content exhibited notably reduced blood glucose concentrations.	[128]
High-amylose wheat	Refined and non-refined high-amylose starch wheat bread.	Human	Compared to the refined low-amylose wheat bread group, the refined high-amylose wheat bread group showed 38% higher fecal butyrate.	[68]
High-amylose wheat	24% and 74% amylose wheat bread	Human	HAS wheat breads showed a 39% lower glycemic response, 24–30% less insulinemic, and incretin responses.	[129]
High-amylose wheat	*sbeII* mutant bread wheat flour	In vitro	High-amylose maize starch showed around 15% lower glycemic response compared to normal-level amylose maize starch.	[130]
High-amylose wheat	>70% high-amylose wheat starch	Sprague-Dawley male rats	Significantly increased cecal pool of total SCFA and individual acids (acetate, propionate, and butyrate), and fecal excretion of SCFA in rats fed the HAS wheat diet than in controls.	[23]
High-amylose rice	Rice contained 7% to 40% amylose	In vitro	Significantly high amounts of butyrate and slightly higher amounts of acetic acid were found in high-amylose rice in vitro fermentation.	[120]
High-amylose rice	High-amylose transgenic rice	Sprague-Dawley Rat	Fecal Acetic acid, propionic acid, and butyric acid were increased significantly. Plasma potassium has significantly increased.	[18]
High-amylose rice	High-amylose rice “Hoshinishiki”.	Human	Significantly lower 24 h blood glucose levels were observed following the consumption of HAS rice.	[102]
High-amylose rice	High-amylose rice “Hoshinishiki”.	Human	Significantly inhibited post-meal blood glucose elevation. Significantly lower blood GLP-1 was observed in the high-amylose rice group after 30–60 min of a meal.	[131]
High-amylose rice	Samgwang (21%) and Dodamssal (47.5%) amylose	C57BL/6J male mice	Serum glucose decreased in both the amylose diet and the high-fat diet but maintained blood glucose levels the same as a normal diet. Significantly reduced plasma GLP-1 compared to HFD but maintained the same level as a normal diet. 47.5% high-amylose rice diet increased significantly higher levels of butyric acid and acetic acid compared to the HFD, 21% amylose rice diet, and the normal diet.	[94]
High-amylose rice	High-amylose-containing YiTang Rice	In vitro	Significantly increased butyrate concentration in high-amylose-containing rice product. Decreased isobutyrate and isovalerate during fermentation compared to inulin and control.	[132]

Note: The study model/species column was included to distinguish human, animal, and in vitro evidence because these study types have different levels of translational strength. Abbreviation: HMG-CoA, 3-hydroxy-3-methylglutary coenzyme A; AIN93G, American Institute of Nutrition 1993 growth purified rodent diet G; C57BL6, C57 black 6 inbred strain of lab mouse; IPA, indole-3-propionic acid; GSH, glutathione; TMAO, trimethylamine N-oxide; OVX, ovearientomized rodent; LDL, low-density lipoprotein; ALT, alanine aminotransferase; HDL, high-density lipoprotein; TG, triglycerides; SCFAs, short-chain fatty acids; VB12, vitamin B12; AmOn, amylose-only barley flour; GIP, glucose-dependent insulinotropic polypeptide; HAS, high-amylose starch; sbeII, starch branching enzyme II; GLP-1, glucagon-like peptide-1; HFD, high-fat diet.

## Data Availability

No new data were created or analyzed in this study. Data sharing is not applicable to this article.

## Abbreviations

The following abbreviations are used in this manuscript:

ALT	Alanine aminotransferase
AmOn	Amylose-only barley flour
CKD	Chronic kidney disease
CRC	Colorectal cancer
CRISPR/Cas9	Clustered regularly interspaced short palindromic repeats/CRISPR-associated protein 9
CTLs	Cytotoxic T lymphocytes
DA	Dopamine
DHA175	Mutagenized durum wheat line
DNA	Deoxyribonucleic acid
DRD1a	Dopamine receptor D1a
DRD2	Dopamine receptor D2
GABA	Gamma-aminobutyric acid
GBA	Gut–brain axis
GBSS	Granule-bound starch synthase
GIP	Glucose-dependent insulinotropic polypeptide
GLP-1	Glucagon-like peptide-1
GPR41	G-protein-coupled receptor 41
GPR43	G-protein-coupled receptor 43
GSH	Glutathione
HAS	High-amylose starch
HFD	High-fat diet
HDL	High-density lipoprotein
IGF-I	Insulin-like growth factor I
IL-1β	Interleukin-1 beta
IL-6	Interleukin-6
LDL	Low-density lipoprotein
miRNAs	MicroRNAs
mRNA	Messenger RNA
NF-κB	Nuclear factor kappa B
OVX	Ovariectomized
PS	High-amylose starch diet
RS	Resistant starch
RS1	Physically inaccessible resistant starch
RS2	Native granular resistant starch
RS3	Retrograded resistant starch
RS4	Chemically modified resistant starch
RS5	Amylose–lipid complex resistant starch
SBE	Starch branching enzyme
SCFA	Short-chain fatty acid
SSIIa	Starch synthase IIa
T2D	Type 2 diabetes
TG	Triglycerides
Th17	T helper 17 cells
TMAO	Trimethylamine N-oxide
TNF-α	Tumor necrosis factor alpha
TS	Low-amylose diet
Treg	Regulatory T cell
